# Study on launch scheme of space-net capturing system

**DOI:** 10.1371/journal.pone.0183770

**Published:** 2017-09-06

**Authors:** Qingyu Gao, Qingbin Zhang, Zhiwei Feng, Qiangang Tang

**Affiliations:** College of Aerospace Science and Engineering, National University of Defense Technology, Changsha, P.R. China; Lanzhou University of Technology, CHINA

## Abstract

With the continuous progress in active debris-removal technology, scientists are increasingly concerned about the concept of space-net capturing system. The space-net capturing system is a long-range-launch flexible capture system, which has great potential to capture non-cooperative targets such as inactive satellites and upper stages. In this work, the launch scheme is studied by experiment and simulation, including two-step ejection and multi-point-traction analyses. The numerical model of the tether/net is based on finite element method and is verified by full-scale ground experiment. The results of the ground experiment and numerical simulation show that the two-step ejection and six-point traction scheme of the space-net system is superior to the traditional one-step ejection and four-point traction launch scheme.

## 1. Introduction

The large accumulation of human-made space debris in orbit is seen as a threat to acquisition and use of space resources in the long term[1,2]. An increasing consensus has been agreed that active debris removal (ADR) is required to ensure long-term sustainability of space resources[3]. Space-net capturing is one of the most promising approaches considered for ADR[4,5]. The disadvantage of space-net capturing is that flexible net dynamics adds more complexity to system design and control. However, the advantage is that the capture process is not constrained by the target debris shape, attitude, and spin rate, thereby reducing the complexity in dealing with an uncooperative target, as stated by Wormnes in literature [6]. The working process of the space-net capturing system can be divided into two processes: deployment and capture. The deploying quality of the net directly determines whether the net could effectively capture the target or not. The deploying quality can be improved by active control method such as space robot tied to the net’s corners [7], however, the system complexity will be inevitably increased. As for the uncontrollable deployment process, the design of the initial launch scheme is extremely important, which is the base for the effective capturing of the space-net system.

In the past few decades, some numerical and experimental studies on space-net capturing system were carried out. Benvenuto and Carta set up a testing facility to characterize, validate, and test a net capture system simulator in a 0-g-affected environment [8–10]. Zhai established a dynamic model of a tether-net system and proposed a capture-error compensation strategy using a feed-forward control method [11,12]. Yu developed a net simulation method based on trivial truss element, which reduced the computational complexity [13]. The relationship between the ejection parameter and deployment quality has been well studied [14–16], and ejection-parameter optimization has been preliminary mentioned [15,17]. The net opening time, maximum area, effective acting time, and effective acting distance were introduced as evaluation indexes for space nets in the literature, which were used to evaluate the deployment performance of the space net.

Modeling and simulating the net structures with a high degree of flexibility and involving large displacements, large deformations, impact loads, and contact dynamics is a very challenging task[18]. The lumped-parameter method is a conventionally used and reliable modeling method for a net structure. Shan introduced the absolute node-coordinate method to solve the space-net modeling problem. The results show that the model based on the absolute-node coordinate method can be consistent with the traditional lumped-parameter method. However, it is computationally expensive[19]. Previous space-net studies mainly focused on one-step ejection and four-point traction launch scheme, which means that four bullets are used to deploy the net through a one-step ejection process. The launch scheme is feasible when the net dimension is relatively small; however, when the net dimension increases to a certain extent, entanglement problem in the net deployment process will occur. We have developed a reduced numerical model to simulate the ejection process in literature[20]. However, more accurate experimental data are still needed to validate this scheme and improve the numerical model.

In the current study, the launch scheme is studied using experiment and simulation, including two-step ejection and multi-point traction analyses. The numerical model of the tether/net is based on the lumped-parameter method and is verified by full-scale ground experiment. The purpose of this paper is to provide the study results of the launch scheme that we obtained from the numerical analysis and ground experiments for the space-net capturing system. This paper is organized as follows. Section 2 gives a brief description of the net launch scheme. Section 3 presents a dynamic modeling approach for the space-net system, and Section 4 introduces the experiments conducted to validate the dynamic model. Section 5 provides a detailed analysis on the launch scheme of the space-net system. Section 6 concludes this paper.

## 2. Launch scheme of space-net capturing system

**Fig 1:**
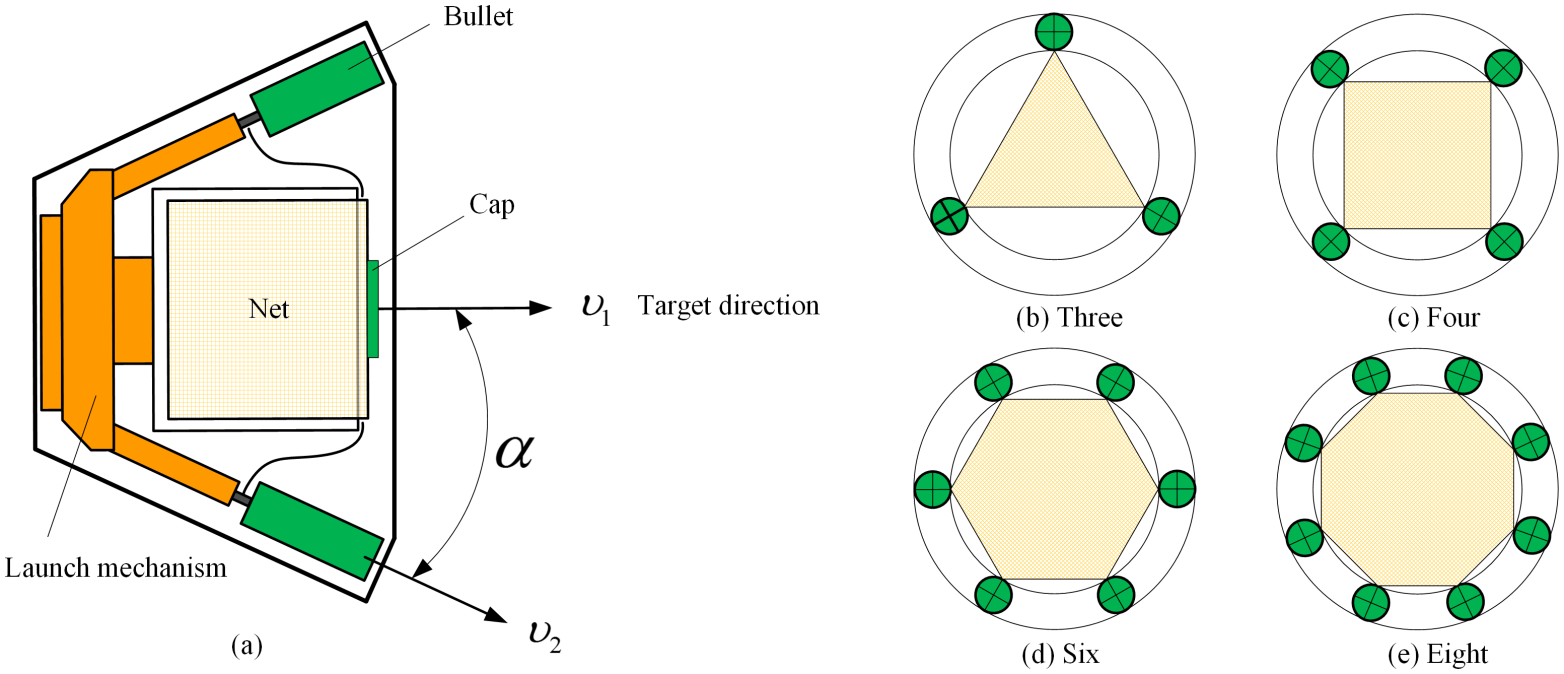
Space-net ejection system.

The conventional launch scheme of the space-net system is introduced in the commonly used one-step ejection and four-point traction mode. In this paper, a two-step ejection and multi-point traction mode is proposed. The space-net launch system designed in our study consists of a flexible net, bullets, a net canister, a canister cap, and the first- and second-stage ejection mechanisms. The flexible net is stowed in the net canister. Each net corner is connected to the bullet, and the center of the net is connected to the canister cap, as shown in Fig 1(a). In the traditional four-point traction mode, the space-net system has four bullets, and the net shape is square, as shown in Fig 1(c). In our study, the three-, six-, and the eight-point traction modes are also investigated using triangular, hexagonal, and octagonal net shapes, as shown in Fig 1(b), 1(d) and 1(e), respectively.

**Fig 2:**
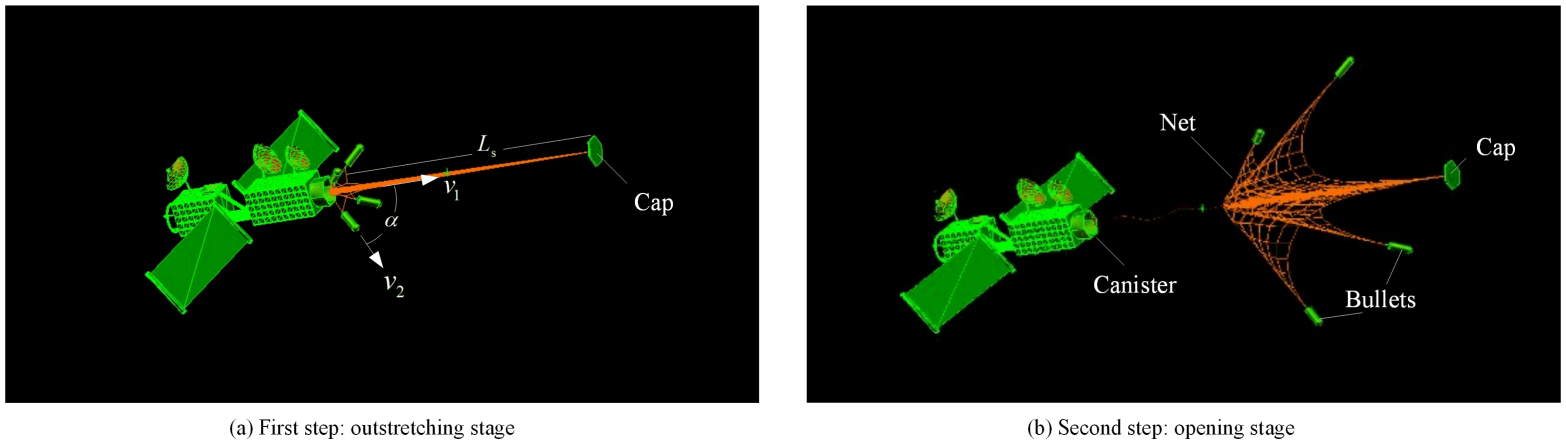
Two- step ejection scheme of the space net.

The ejection process is divided into two successive steps, namely, the outstretching stage in the first step and the opening stage in the second step. In the outstretching stage shown in Fig 2(a), the canister cap attains outstretching velocity *v*_1_ from the first-stage ejection mechanism. Simultaneously, the net is outstretched under the traction of the cap. In the opening stage shown in Fig 2(b), when the cap is ejected to ignition length *L*_s_, the bullets attain velocity *v*_2_ and ejection angle *α* from the second-stage ejection mechanism, and the net is deployed to its fully deployed shape from the back forward. Ejection angle *α* is defined as the angle between the velocity direction of the bullet and the direction of the canister axis.

## 3. Modeling methodology

### 3.1. Equation of motion

**Fig 3:**
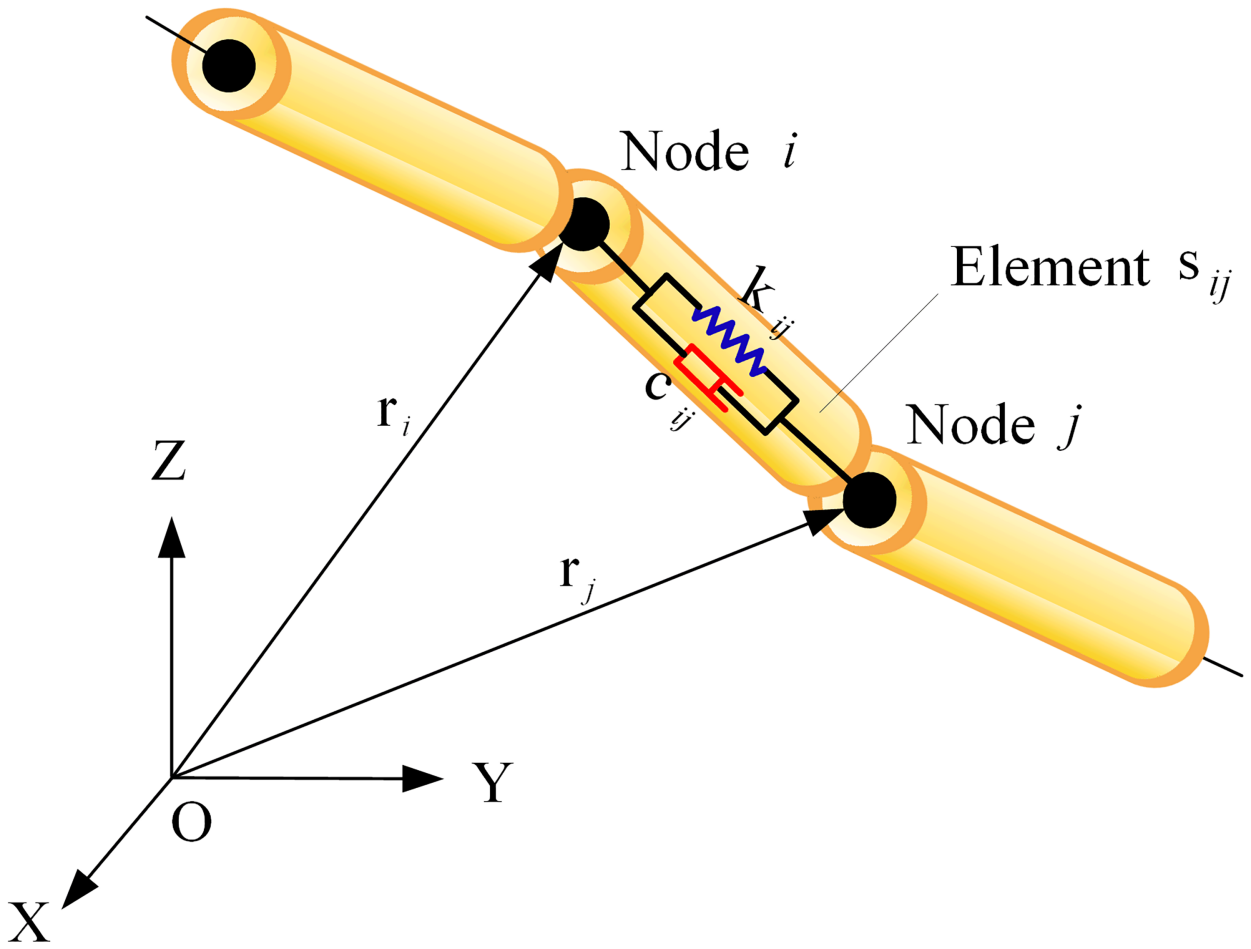
Finite element model of the tether element.

Considering computational efficiency, the space-net launch system is modeled using the lumped-parameter method. In this dynamic model shown in Fig 3, the tether element is equivalent to the combination of a semi-spring and a damper, and the mass of the element is evenly distributed at the two end nodes.

**Fig 4:**
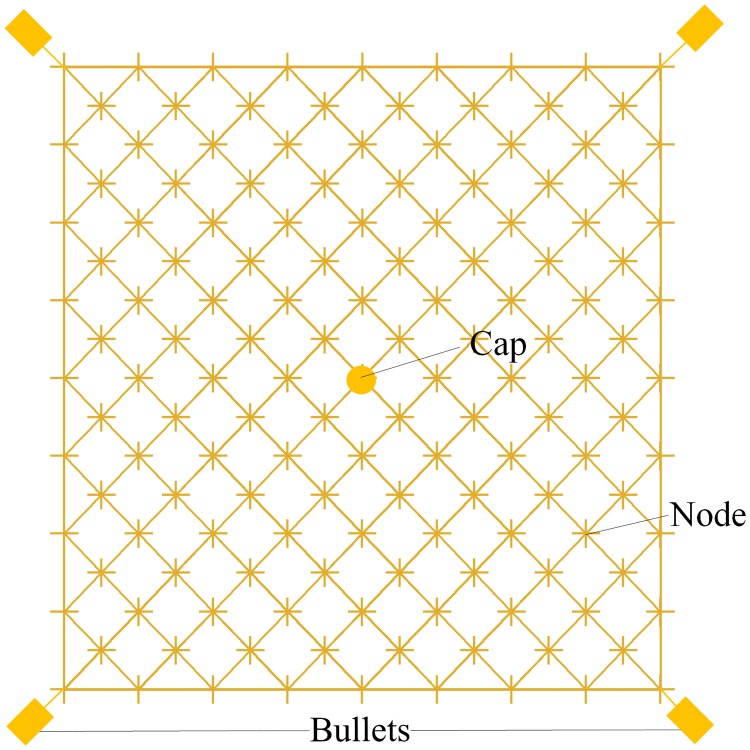
Multi-body system model of the net.

The tether element that connects nodes *i* and *j* is defined as element *s*_*ij*_. The net is modeled with the tether elements according to its actual grid mesh, as shown in Fig 4. Moreover, each node is modeled as a point mass with three translational degrees of freedom. Similarly, the cap and bullets are modeled as point masses. Consequently, the flexible net system is considered as a multi-body system subjected to elastic and aerodynamic forces and gravity. The equations of motions of the *i*th tether node can be expressed as

$$m_{i}{\ddot{r}}_{i}=\sum_{j\in \Omega \{i\}}T_{ij}+\sum_{j\in \Omega \{i\}}\frac{1}{2}G_{ij}+\sum_{j\in \Omega \{i\}}\frac{1}{2}F_{ij}^{D}+\sum_{j\in \Omega \{i\}}\frac{1}{2}F_{ij}^{L}$$ (1)

where Ω{*i*} is the defined node ID space, which is a collection of the nodes in element *s*_*ij*_ that share node *i*. All equations of motion for the flying weights and tether nodes are nonlinear.

### 3.2. Elastic forces

The elastic forces of the tether element are produced by the spring and damper characteristics of the tether. These forces are parallel to the direction of the tether element. Certain circumstances occur when the tether loses tension. However, the tether cannot sustain compression forces. The elastic force in element *s*_*ij*_ is thus given by

$$T_{ij}=\{\begin{array}{cc} A_{ij}\sigma (\varepsilon_{ij})+2\zeta \sqrt{\rho_{lij}EA_{ij}}{\dot{l}}_{ij} & l_{ij}>l_{ij}^{0}\\ 0 & l_{ij}\leq l_{ij}^{0} \end{array}$$ (2)

where *σ*(*ε*_*ij*_) denotes the stress–strain relationship and *ζ* is the damping constant of the tether element.

### 3.3. Gravity

The gravitational force acting on node *i* can be obtained by

$$G_{ij}=-\mu M_{e}m_{i}\frac{r_{i}}{{\left|r_{i}\right|}_{}^{3}}$$ (3)

where *μ* is the universal gravitational constant, *M*_*e*_ is the mass of the Earth, *m*_*i*_ is the mass distributed to node *i*, and *r*_*i*_ is the position vector.

### 3.4. Aerodynamic forces

The aerodynamic forces acting on the tether element can be determined from the cross-flow principle. Each tether element is considered to be an ideal cylinder with no porosity. The aerodynamic lift and drag coefficients of element *l*_*ij*_ are defined as functions of attack angle *α*_*ij*_. Lift and drag coefficients $C_{ij}^{D}$ and $C_{ij}^{L}$, respectively, of an inclined cylinder are given as follows[21]:

$$\begin{array}{l} C_{ij}^{D}=C_{ij}^{f}+C_{ij}^{b}{\text{sin}}^{3}\alpha_{ij}\approx 0.022+1.1{\text{sin}}^{3}\alpha_{ij}\\ C_{ij}^{L}=C_{ij}^{b}{\text{sin}}^{2}\alpha_{ij}\text{cos}\alpha_{ij}\approx 1.1{\text{sin}}^{2}\alpha_{ij}\text{cos}\alpha_{ij} \end{array}$$ (4)

where $C_{ij}^{f}$ and $C_{ij}^{b}$ denote the skin-friction and cross-flow drag coefficients, respectively. Hence, the drag and lift vectors can be expressed as

$$\begin{array}{l} F_{ij}^{D}=\frac{1}{2}\rho^{air}C_{ij}^{D}d_{ij}l_{ij}^{0}{\left\|\upsilon_{ij}^{r}\right\|}^{2}e_{ij}^{D}\\ F_{ij}^{L}=\frac{1}{2}\rho^{air}C_{ij}^{L}d_{ij}l_{ij}^{0}{\left\|\upsilon_{ij}^{r}\right\|}^{2}e_{ij}^{L} \end{array}$$ (5)

where *ρ*_air_ is the density of air, *d*_*ij*_ is the diameter of the tether, $\upsilon_{ij}^{r}$ is the wind velocity of the center of element *l*_*ij*_, and $e_{ij}^{D}$ and $e_{ij}^{L}$ are the drag and lift vectors, respectively.

To improve the solution efficiency and ensure computational accuracy, the equation of motion is integrated into the FORTRAN language using the variable-step-size Runge–Kutta method.

## 4. Experimental design and model validation

The stress–strain relationship and the damping coefficient of the tether were obtained from the static tensile and ball-falling tests. The dynamic model of the space-net system is verified by the airdrop experiments.

### 4.1. Static tensile test

**Fig 5:**
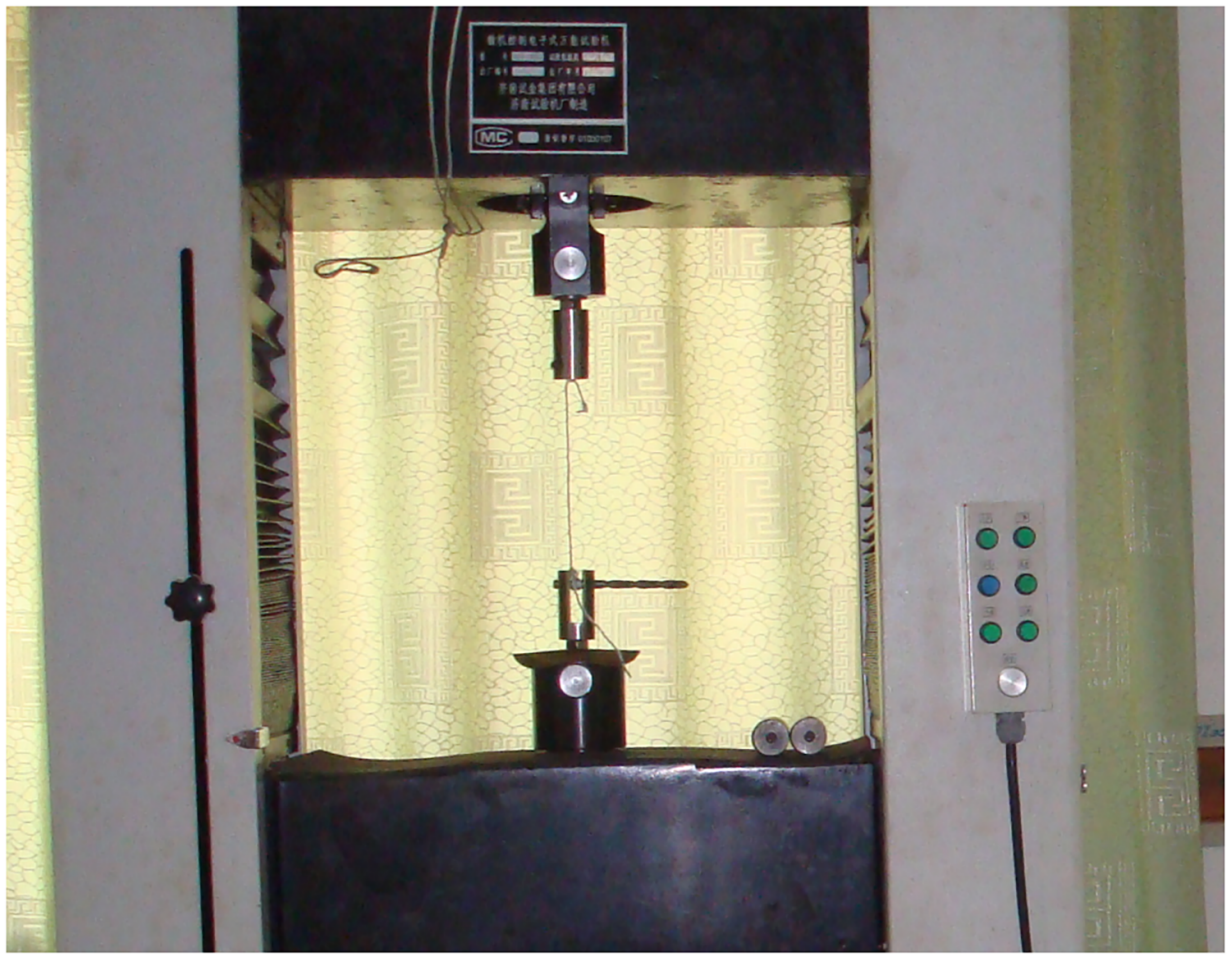
Tether static tensile test.

**Fig 6:**
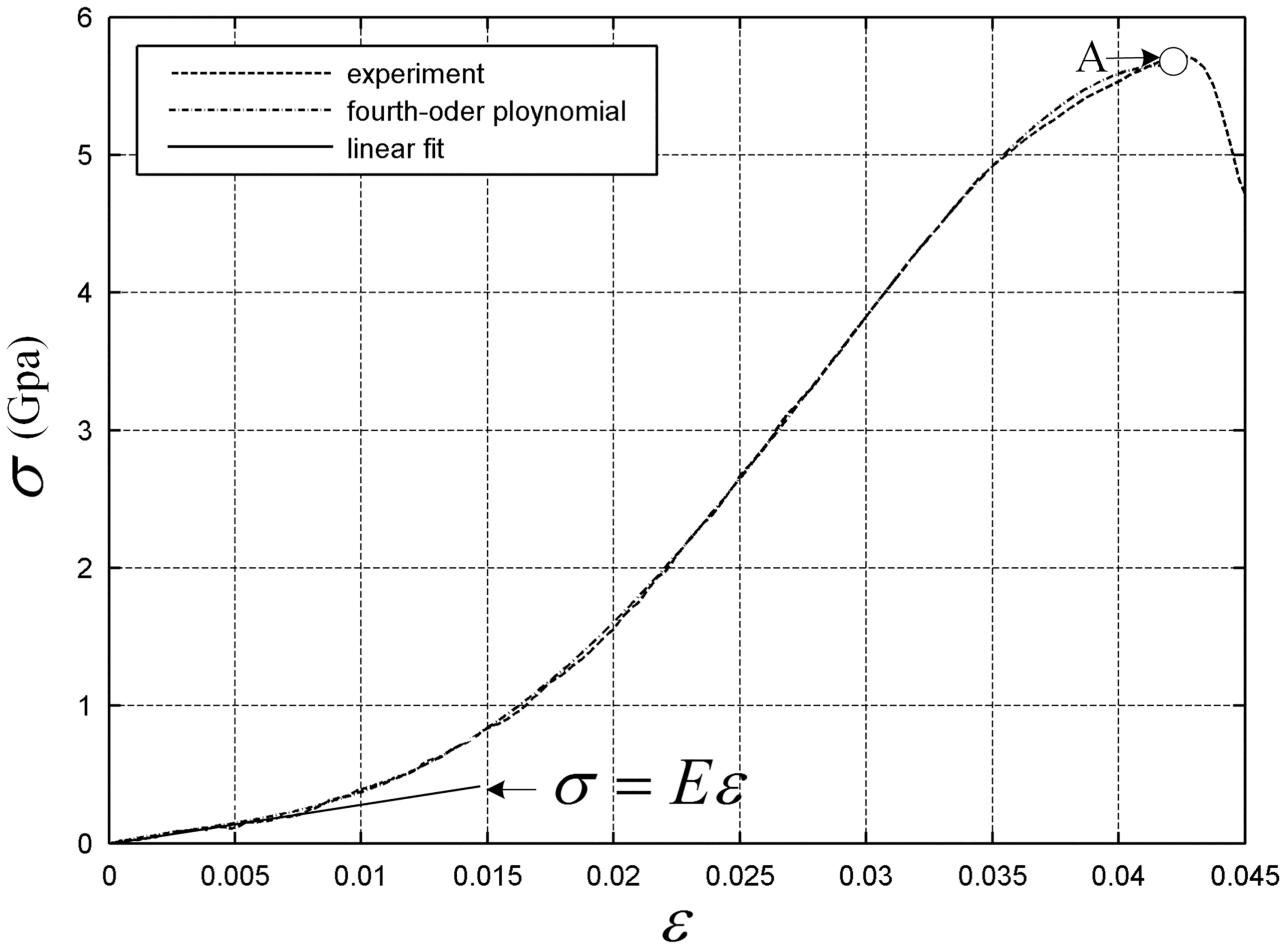
Tether stress–strain curve.

Previous studies tend to treat the stress–strain relation according to the tether linear hypothesis. However, in reality, the tether has strong nonlinear features. Carrying out a tether static tensile test is necessary to obtain accurate stress–strain relationships, as shown in Fig 5. From the stress–strain curve shown in Fig 6, we can conclude that the linear hypothesis is satisfied in a small range of strain, and strong nonlinear features appear when the strain is beyond this range. Because the force in the tether of the space net is always less than the point value of the circle in the test curve(see S1 Table), we choose the test curve before point A to define stress–strain relationship function *σ*(*ε*_*ij*_), which can be substituted with a fourth-order polynomial curve.

### 4.2. Ball-falling test

**Fig 7:**
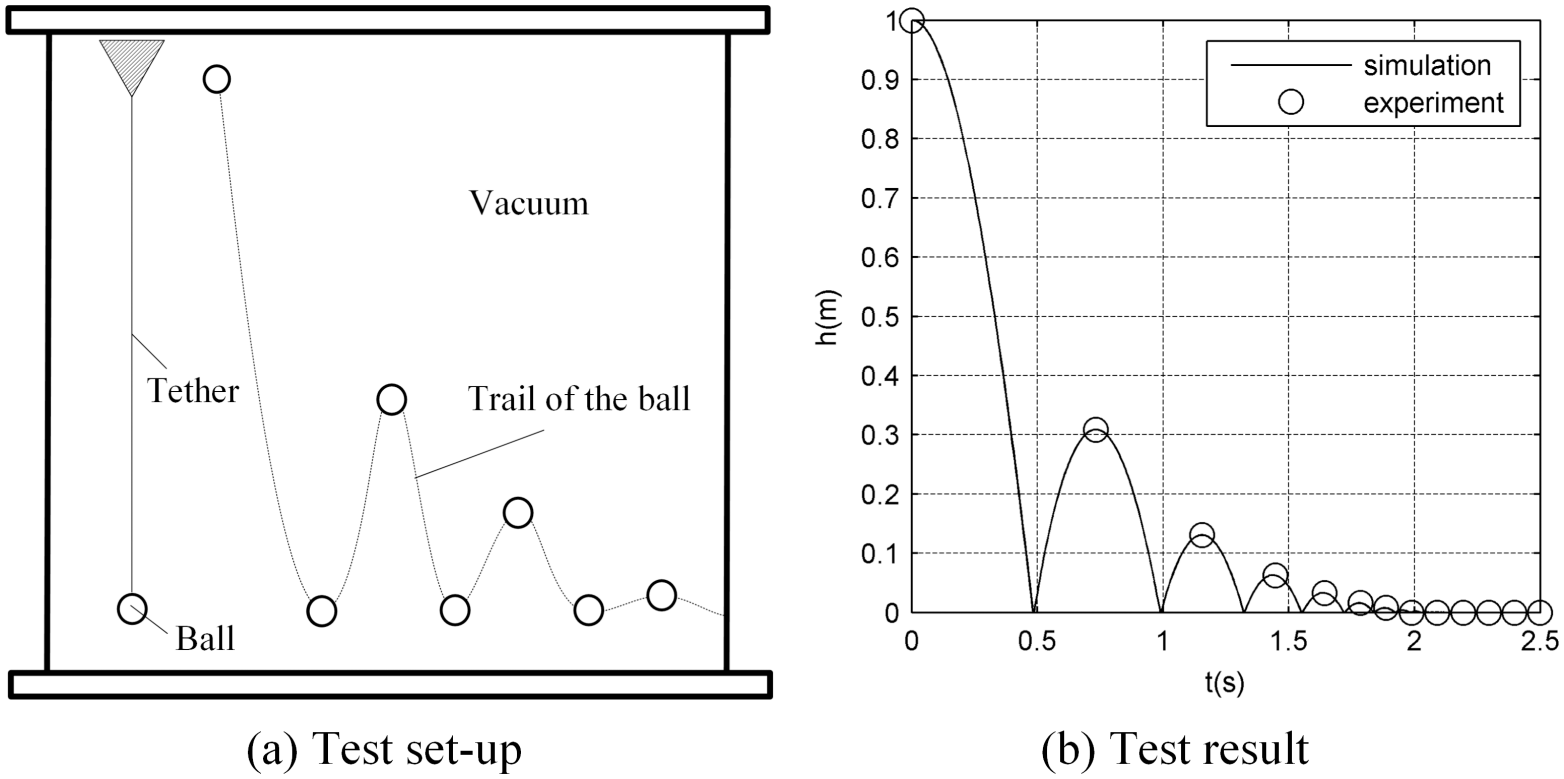
Test of the tether-damping constant.

The damping constant of the tether is obtained from a ball-falling test. Fig 7(a) shows that one end of the test tether is fixed and the other end is tied to a ball. Once the ball vertically falls, the camera will record the frequency and amplitude behavior of the ball-falling system. Because the elastic force and gravity of the falling-ball system can be obtained from Eqs (2) and (3), the damping constant can be determined. Fig 7(b) shows that if the damping constant is properly set, the experimental and numerical simulation results will show a good consistency (see S2 Table).

### 4.3. Full-scale airdrop experiments

**Fig 8:**
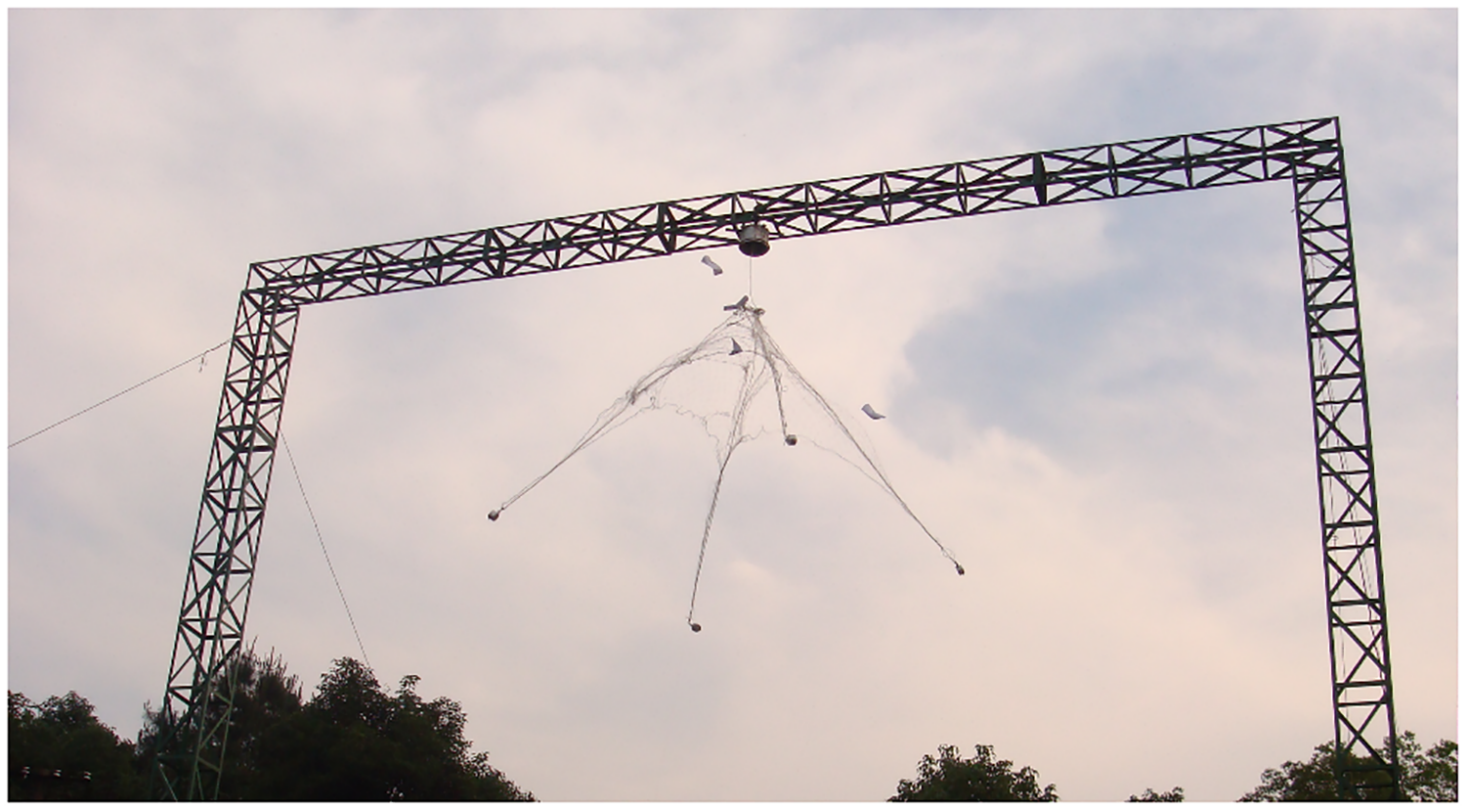
Experimental setup of the airdrop tests.

Full-scale airdrop experiments were conducted to verify the numerical model presented in Section III, as is shown in Fig 8. The experimental system consists of the control platform, ejector, flexible net, and high-speed cameras. The ejection direction is vertically downward. In the ground experiments, the position information of the bullets is accurately obtained using high-speed cameras (Table A-C in S3 Table).

### 4.4. Model validation

The dynamic model of the space-net system is verified by the full-scale airdrop experiments. The validation experiments consists of three test cases, as is descript in Table 1. The net is meshed with rhombic grids and the grid size 0.4 m. Kevlar 49 (Density *ρ* = 1.44 *g*/*cm*^3^, Modulus of elasticity *E* = 200 Gpa) is selected as material of the nets. The mass of bullet is set 1.5 kg.

**Table 1:** Test cases in the validation experiments.

Case	Net shape	Net dimension	Ejection velocity	Bullets
1	square	40 m	20 m/s	1.5 kg×4
2	square	40 m	25 m/s	1.5 kg×4
3	hexagon	20 m	25 m/s	1.5 kg×6

**Fig 9:**
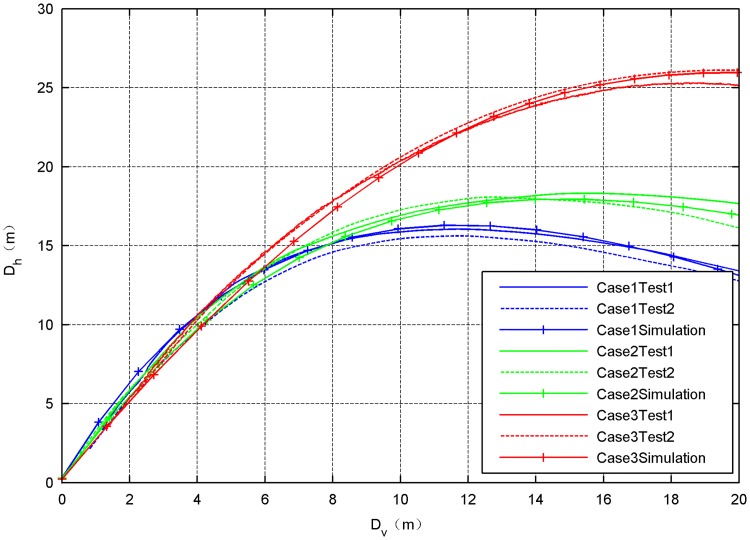
Deployment performances of the space net in validation tests.

**Fig 10:**
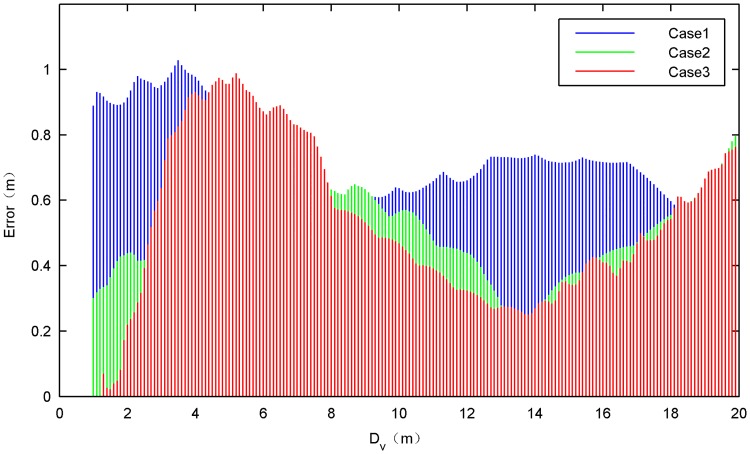
Errors between the simulation result and test result.

The horizontal distance *D*_*h*_ between the diagonal bullets and the vertical descent distance *D*_*v*_ of the bullets are selected to describe the deployment performance of the space net. Fig 9 shows the the results obtained from numerical model and the ground experiments. And Fig 10 shows the errors between simulations and tests.

In the test cases, the ejection velocity, the net shape and the net dimension are set to different values. It can be seen that all the simulation results of those three cases has good consistency with the test results. The error between the simulation and the test is less than 1 m and the relative value is less than 10%. As three is a certain degree of uncertainty in the net deployment process, we think that the model accuracy is acceptable and can be used to describe the development process of the space net in the ground environment.

## 5. Results and discussion

### 5.1. Two-step ejection mode

**Fig 11:**
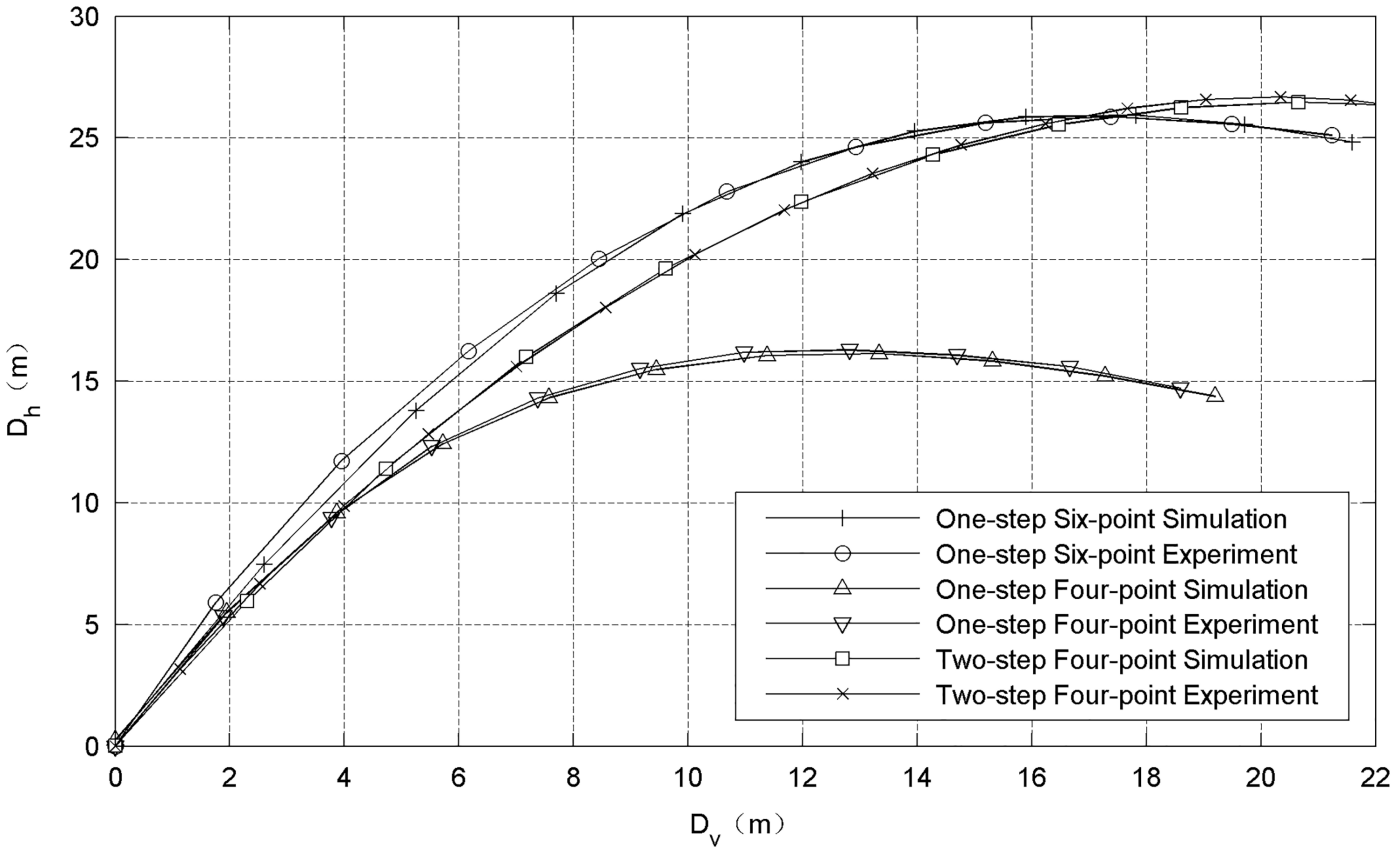
Deployment radius versus flight distance curve in the airdrop test.

Fig 11 shows the differences in the deployment radius between the one-step and two-step ejection modes. The deployment radius in the two-step ejection mode is obviously better than that of the one-step scheme, which shows the superiority of the two-step scheme.

**Fig 12:**
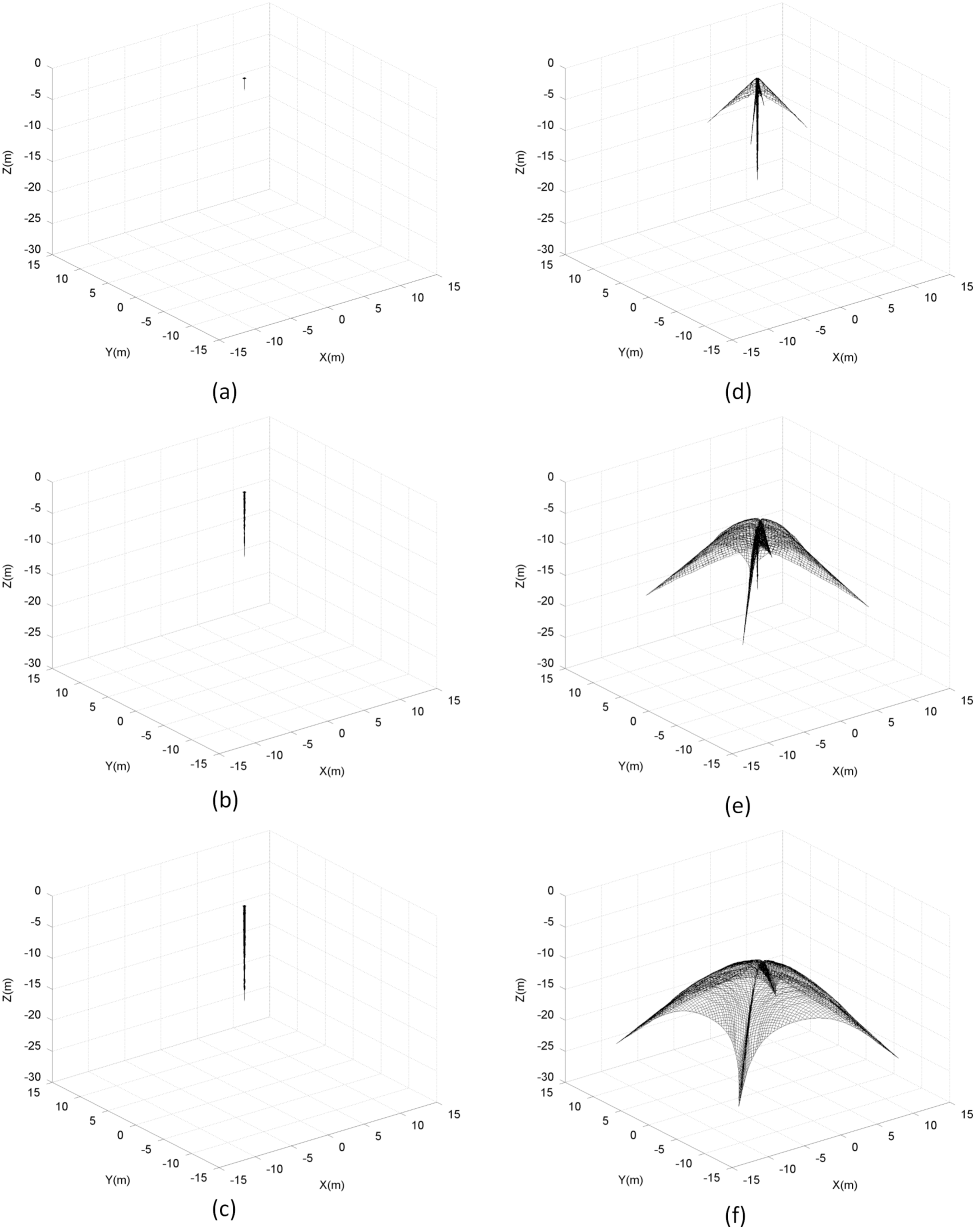
Shape of space net during deployment.

The shape of the net in the two-step deployment scheme is shown in Fig 12. In this ejection mode, the launch process is divided into two independent stages, controlled step by step and in an orderly manner, where the net tethers are effectively segregated. In this mode, penetration and entanglement problems would less probably occur, as indeed shown by the experimental results.

The results also show that the mass distribution and initial-velocity loading condition of the net capturing system play an important role in the deployment process. In the two-step mode, the cap and the bullets are added as lump masses into the center and corner points of the lightweight net, and the initial velocities are established by the two ejection mechanisms. The relationship between the initial velocity at the center and corner points directly affects the performance of the net during the deployment process. When a difference exists, the momentum of the net system will experience redistribution subject to the elastic forces of the tether, accompanied with net-shape change and system energy dissipation. Through the setting of the initial two-step ejection condition, we can enhance the ability of the net configuration control during the deployment process.

### 5.2. Multi-point traction mode

**Fig 13:**
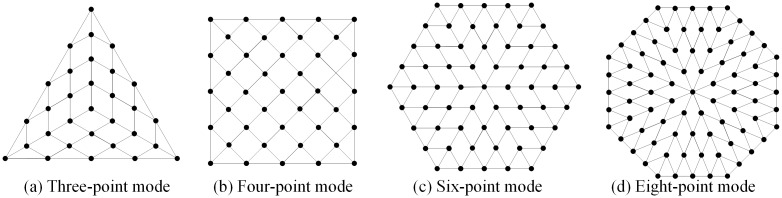
Rhombic nets used in different traction modes.

The results of the airdrop tests shown in Fig 11 also indicate that the six-point traction mode of the space-net system is better than the traditional four-point traction mode. A series of numerical simulations were carried out to further analyze the differences among the different traction modes. Fig 13 shows that rhombic nets with mass of 2.0 kg is chosen in these simulations. The dimension between the diagonal points of the nets (design diameter *D*_*d*_) is 56 m, and the size of the grid is chosen to be 0.4 m. The selected material for the nets is Kevlar 49. Initial velocity *v*_0_ of the flying weights is set to 15 m/s, and ejection angle *α* is set to 25°. To ensure that the net systems under different traction modes have the same initial mechanical energy, the masses of the flying weights are set according to the list in Table 2.

**Table 2:** Number and mass of bullets under different traction modes.

Traction mode	Mass of net[kg]	Number of bullets	Mass of bullets [kg]
3	2.0	3	2
4	2.0	4	1.5
6	2.0	6	1
8	2.0	8	0.75

The simulation results of the four types of traction modes are shown in Figs 14–21. Because the nets in these four simulations share the same design diameter *D*_*d*_, we analyze the results from the other two aspects: flight and mechanical performance aspects.

**Fig 14:**
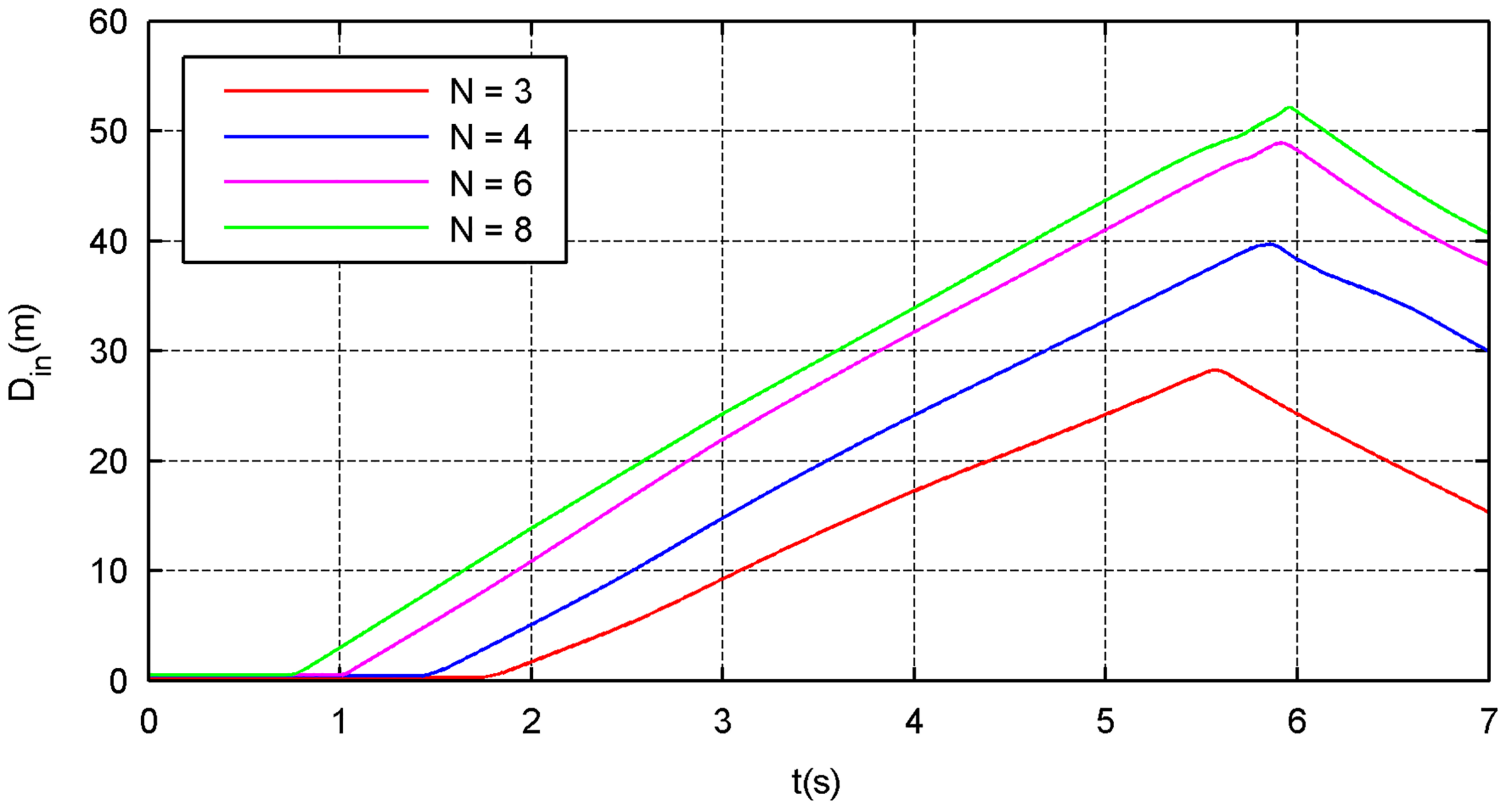
Internal-diameter curve.

**Fig 15:**
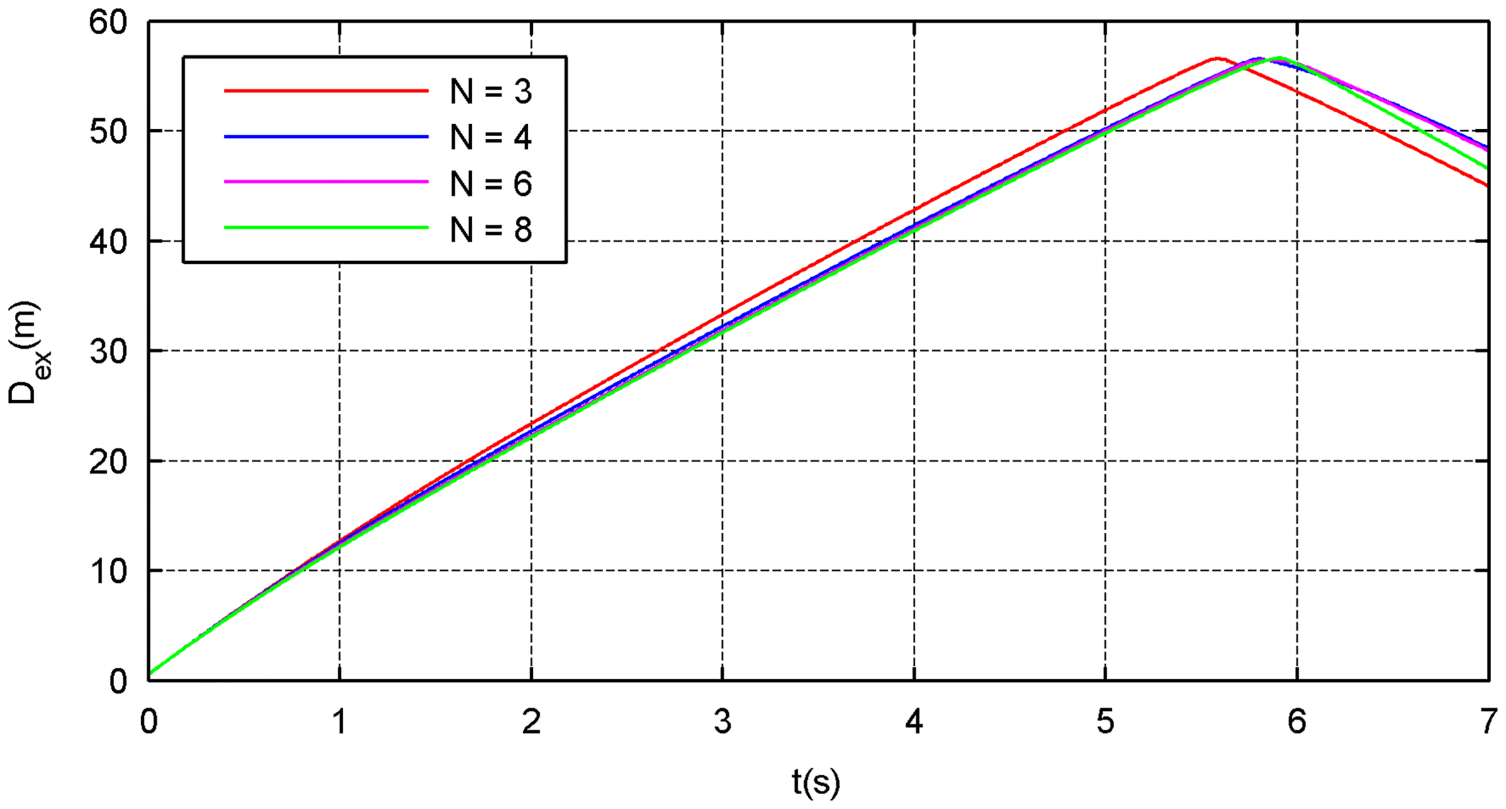
External-diameter curve.

**Fig 16:**
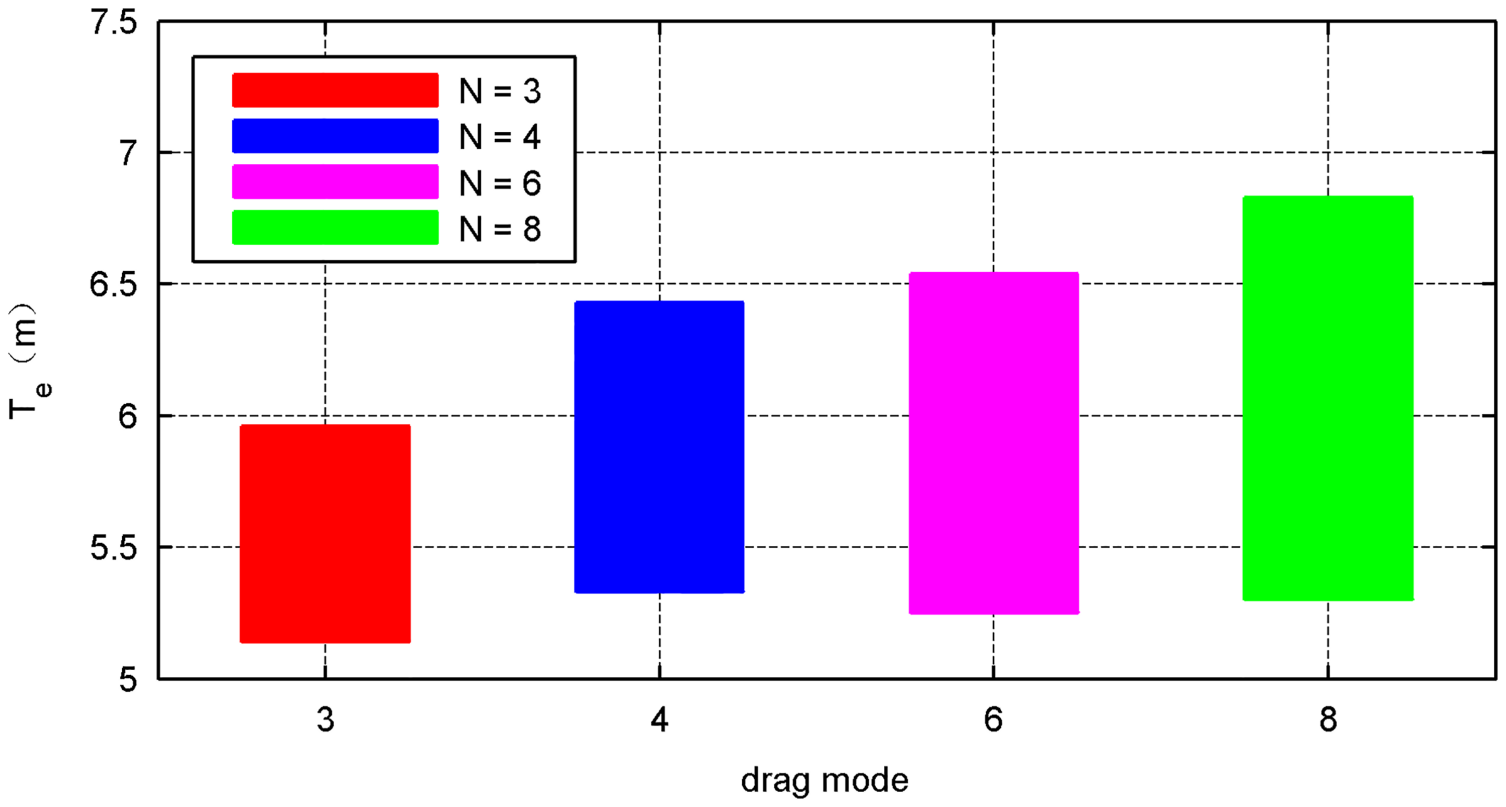
Effective time curve.

**Fig 17:**
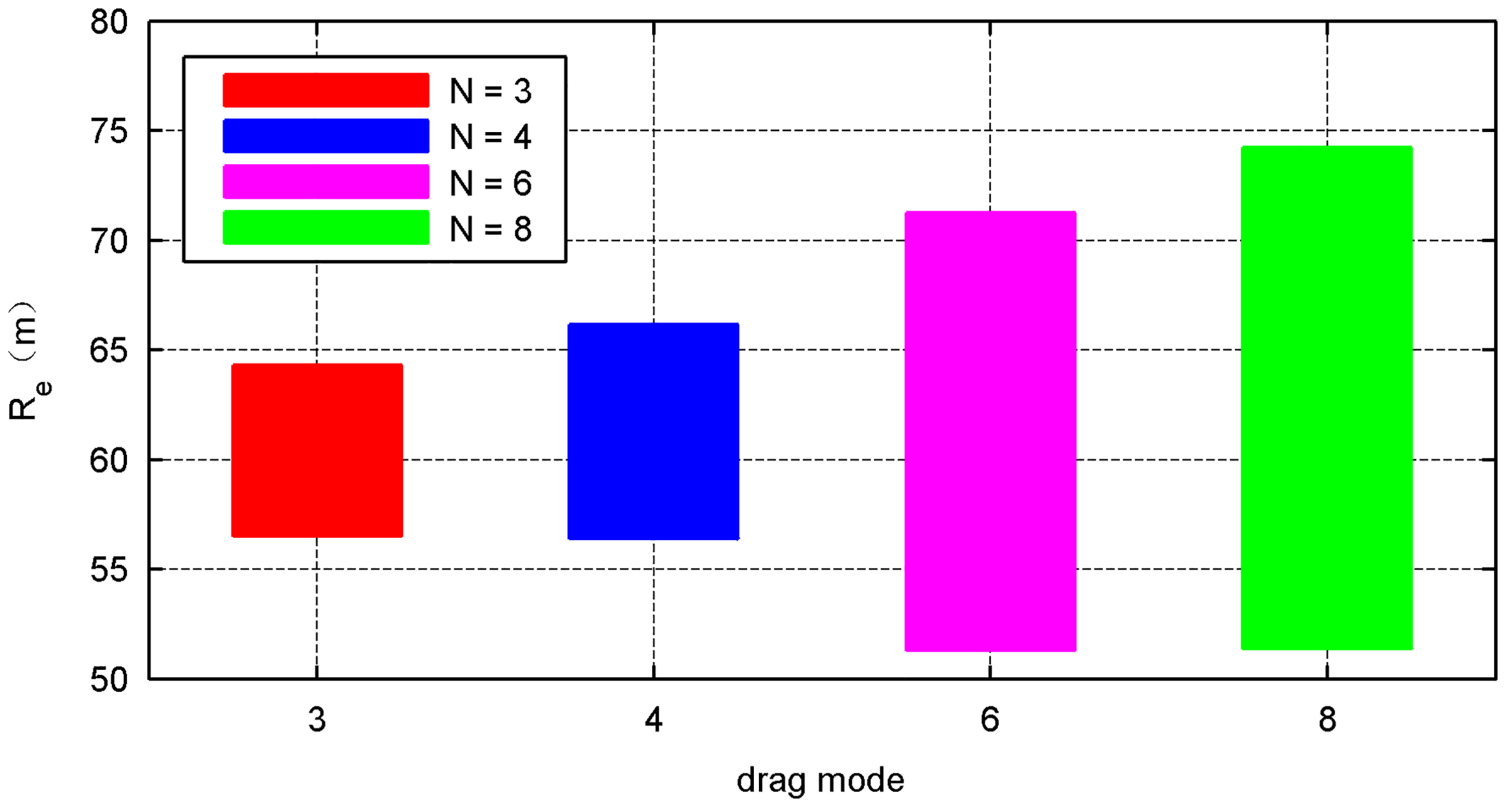
Effective distance curve.

**Fig 18:**
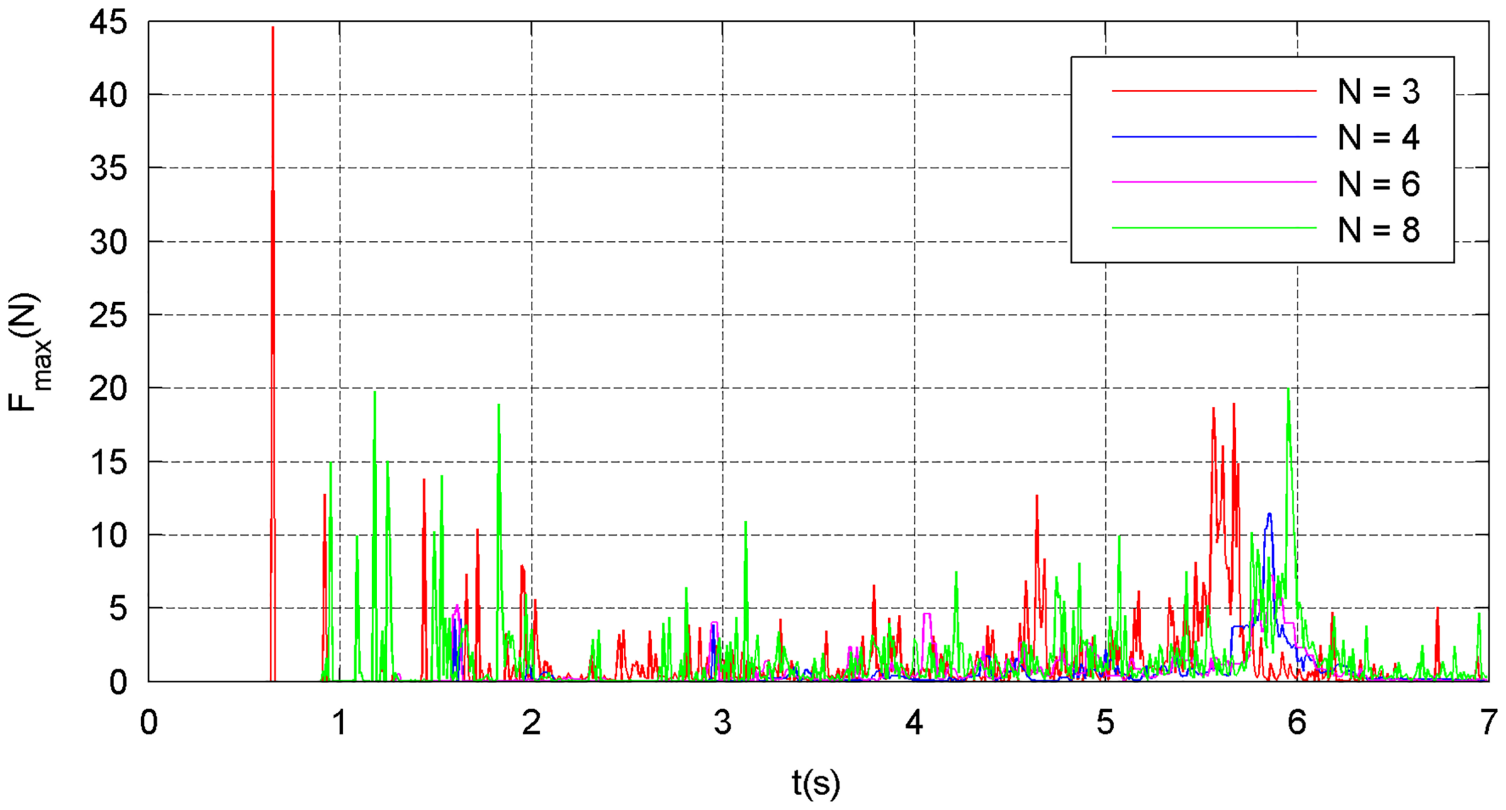
Maximum internal-force curve.

**Fig 19:**
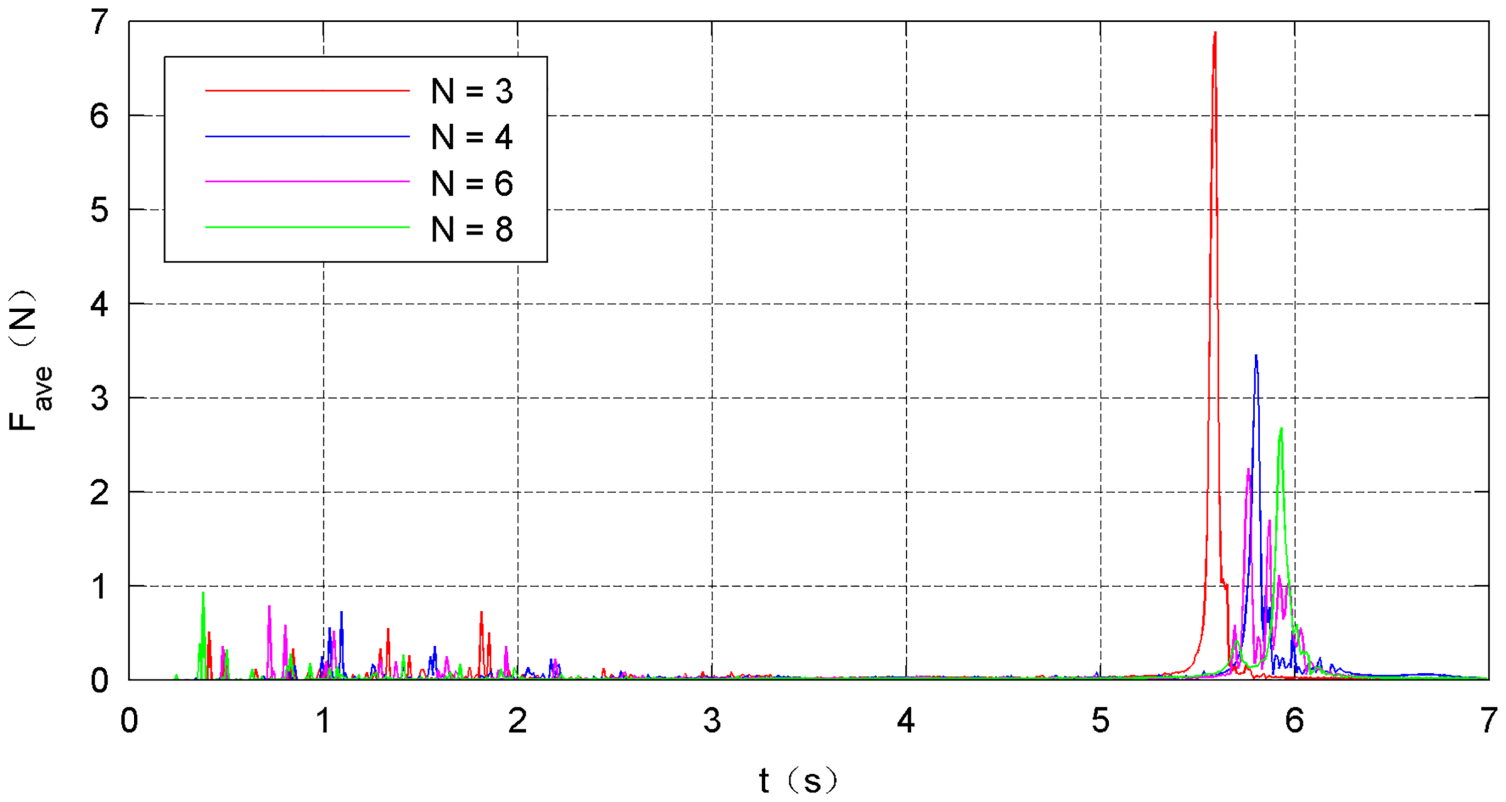
Average internal-force curve.

**Fig 20:**
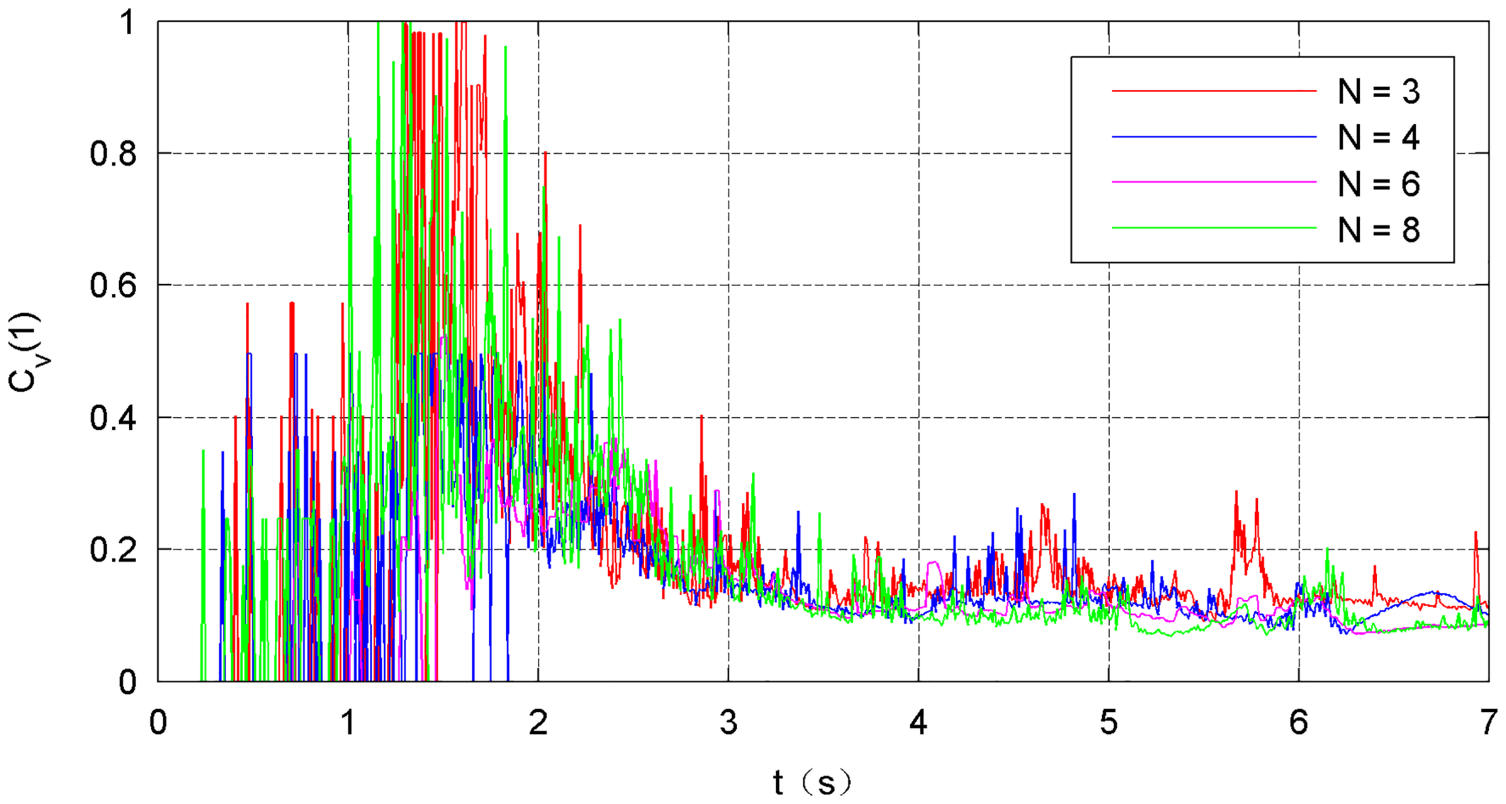
Variation in the internal-force curve.

**Fig 21:**
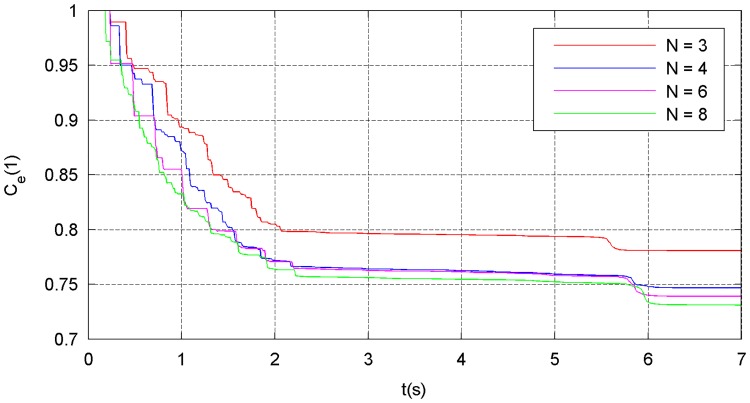
Energy-attenuation curve.

Figs 14 and 15 show that all internal- and external-diameter indexes in the four simulations achieve the design value, which means that the eight-point net achieve the maximum projection area. However, a slight difference appears in the time required to reach the maximum area in which a larger number of traction points require a longer time. Figs 16 and 17 show the effective time and effective distance of the space net, which stand for the time and distance consumed during the effective deployment. Both the effective time and effective distance indexes increase with the increase in the traction points; however, they are considerably higher in the six- and eight-point modes than those in the three- and four-point modes.

Figs 18–21 show the mechanical performance of the nets under different traction modes. The maximum, average, and variance in the internal forces decrease as the number of traction points increases. However, the energy attenuation exhibits an opposite trend. The results of the mechanical performance indexes illustrate that a larger number of traction points are advisable from the point of view of the strength tolerance aspect of the net. In contrast, when energy is taken into account, a smaller number of traction points should be chosen.

All these simulations highlight that selecting an appropriate number of traction modes for the space-net launch system is important. An increase in the traction-point number provides the following benefits: (a) increase in the deployment degree, which helps reduce system sensitivity for other parameters such as ejection angle and speed; further, the system capturing capability is effectively improved, and (b) a more even distribution of the internal force of the net, which minimizes the volatility and tangling possibility of the net itself. The disadvantages mainly lie on the following two points: (a) increase in the energy dissipation of the space-net system, thus reducing the energy utilization rate, and (b) increase in system complexity, which reduces the system reliability.

## 6. Conclusion

Experimental observations and numerical simulations have been conducted to investigate the launch scheme of a space-net system. The numerical model of a space-net system was developed using the semi-spring–mass damp methods. Full-scale ground experiments were performed to validate the numerical model and test the deployment quality of the space net under different launch schemes. Both the ground experiment and numerical simulation results showed that the step-by-step ejection method is effective in solving the entanglement problem during the net deployment process. In addition, the multi-point traction mode of the space-net launch system could provide a better deployment performance than the traditional traction mode. Considering the requirement in engineering practice, we believe that the two-step ejection and six-point traction launch scheme could be a better improvement to the traditional one-step ejection and four-point traction launch scheme of the space-net capturing system.

## Supporting information

S1 Table Result of the static tensile test. (XLSX)

S2 Table Result of the ball-falling test. (XLSX)

S3 Table Result of the airdrop test. (XLSX)

## References

[1] Imburgia JS. Space debris and its threat to national security: a proposal for a binding international agreement to clean up the junk. Vanderbilt Journal of Transnational Law. 2011; 44: 589–641.

[2] Pelton JN. Space debris and other threats from outer space. Arlington: Springer; 2013.

[3] Bonnal C, Ruault JM, Desjean MC. Active debris removal: Recent progress and current trends. Acta Astronautica. 2013;85(0):51–60.

[4] Shan M, Jian Guo, Eberhard Gill. Review and comparison of active space debris capturing and removal methods. Progress in Aerospace Sciences. 2016;80(2016):18–22.

[5] Wormnes K, Letty RL, Summerer L, Schonenborg R, Dubois-Matra O, Luraschi E, et al. ESA technologies for space debris remediation. 6th European Conference on Space Debris; 2013; Darmstadt.

[6] Wormnes K, Jong JHD, Krag H, Visentin G. Throw-nets and tethers for robust space debris capture. International Astronautical Congress; 2013; Beijing.

[7] Huang P, Hu Z, Fan Z. Dynamic modelling and coordinated controller designing for the manoeuvrable tether-net space robot system. Multibody System Dynamics. 2015:115–41.

[8] Benvenuto R, Salvi S, Lavagna M. Dynamics analysis and GNC design of flexible systems for space debris active removal. Acta Astronautica. 2015;110:247–65.

[9] Benvenuto R, Lavagna M. Flexible capture devices for medium to large debris active removal: simulations results to drive experiments. 12th Symposium on Advanced Space Technologies in Automation and Robotics; 2013; Noordwijk.

[10] Benvenuto R, Carta R. Active debris removal system based on tethered-nets: experimental results. 9th Pegasus-AIAA Student Conference; 2013; Politecnico di Milano.

[11] Zhai G, Qiu Y, Liang B, Li C. On-orbit capture with flexible tether–net system. Acta Astronautica. 2009;65:613–23.

[12] Zhai G, Qiu Y, Liang B, Li C. System dynamics and feedforward control for tether-net space robot system. International Journal of Advanced Robotic Systems. 2009;6: 137–144.

[13] Yu Y, BaoYin HX, Li JF. Dynamic modelling and analysis of space webs. Science China Physics, Mechanics & Astronomy. 2011; 54: 783–791.

[14] Salvi S. Flexible devices for active space debris removal: the net simulation tool. M. Eng. Thesis, Politecnico di Milano. 2014.

[15] Chen Q, Yang LP. Research on casting dynamics of orbital net systems. Journal of Astronautics. 2009; 30(5): 1829–33.

[16] Zhai G, Zhang JR, Yao Z. Circular orbit target capture using space tether-net system. Mathematical Problems in Engineering. 2013; 2013(4); 87–118.

[17] Li JY, Yu Y, Baoyin HX. Projecting parameters optimization for space web systems. Journal of Astronautics. 2012;33(6):823–9.

[18] Benvenuto R, Salvi S, Lavagna M. Dynamics analysis and GNC design of flexible systems for space debris active removal. Acta Astronautica. 2015;110:247–65.

[19] Shan MH, Guo J, Gill E. Deployment dynamics of tethered-net for space debris removal. Acta Astronautica. 2017; 132: 293–302.

[20] Gao QY, Tang QG, Zhang QB, Feng ZW. Dynamics analysis of a two-stage projection scheme of space nets system. ACTA ARMAMENTARII. 2016; 37: 619–726.

[21] Chen Q. Design and Dynamics of an Orbital Net-Capture System. Ph. D. Thesis, National University of Defense Technology. 2010.

